# Mechanical power normalized to lung-thorax compliance predicts prolonged ventilation weaning failure: a prospective study

**DOI:** 10.1186/s12890-021-01566-8

**Published:** 2021-06-17

**Authors:** Alessandro Ghiani, Joanna Paderewska, Swenja Walcher, Claus Neurohr

**Affiliations:** 1 Department of Pulmonology and Respiratory Medicine, Schillerhoehe Lung Clinic (affiliated to the Robert-Bosch-Hospital GmbH, Stuttgart), Solitudestrasse 18, 70839 Gerlingen, Germany; 2 German Center for Lung Research (DZL), München, Germany

**Keywords:** Mechanical ventilation, Ventilator weaning, Tracheostomy

## Abstract

**Background:**

Mechanical power (MP) of artificial ventilation, the energy transferred to the respiratory system, is a chief determinant of adequate oxygenation and decarboxylation. Calculated MP, the product of applied airway pressure and minute ventilation, may serve as an estimate of respiratory muscle workload when switching to spontaneous breathing. The aim of the study was to assess MP’s discriminatory performance in predicting successful weaning from prolonged tracheostomy ventilation.

**Methods:**

Prospective, observational study in 130 prolonged mechanically ventilated, tracheotomized patients in a specialized weaning center. Predictive weaning outcome ability of arterial blood gas analyses and indices derived from calculated MP at beginning and end of weaning was determined in terms of area under receiver operating characteristic curve (AUROC) and measures derived from k-fold cross-validation (likelihood ratios, diagnostic odds ratio, F_1_ score, and Matthews correlation coefficient [MCC]).

**Results:**

Forty-four (33.8%) patients experienced weaning failure. Absolute MP showed poor discrimination in predicting outcome; whereas specific MP (MP normalized to dynamic lung-thorax compliance, LTC_dyn_-MP) had moderate diagnostic accuracy (MCC 0.38; AUROC 0.79, 95%CI [0.71‒0.86], p < 0.001), further improved by correction for corresponding mechanical ventilation P_a_CO_2_ (termed the power index of the respiratory system [PI_rs_]: MCC 0.52; AUROC 0.86 [0.79‒0.92], p < 0.001). Diagnostic performance of MP indices increased over the course of weaning, with maximum accuracy immediately before completion (LTC_dyn_-MP: MCC 0.49; AUROC 0.86 [0.78‒0.91], p < 0.001; PI_rs_: MCC 0.68; AUROC 0.92 [0.86‒0.96], p < 0.001).

**Conclusions:**

MP normalized to dynamic lung-thorax compliance, a surrogate for applied power per unit of ventilated lung volume, accurately discriminated between low and high risk for weaning failure following prolonged mechanical ventilation.

**Supplementary Information:**

The online version contains supplementary material available at 10.1186/s12890-021-01566-8.

## Background

Weaning from prolonged tracheostomy ventilation is often seen as an art rather than a scientifically based, standardized process. The main reason may be that these patients have so far attracted little attention from scientists, resulting in a lack of evidence-based recommendations, with significant differences in approaches at dedicated weaning and home ventilation centers [1‒3]. Furthermore, predictors of weaning and extubation outcome have so far been predominantly validated in patients following short-term mechanical ventilation [4]. Diagnostic accuracy of such predictors (e.g. maximum inspiratory pressure, minute ventilation, respiratory rate) varies considerably, perhaps due to limited agreement between these parameters and the underlying pathophysiology of weaning failure, in prolonged ventilated patients namely an imbalance between work of breathing and capacity of respiratory muscles [5, 6]. The mechanical power (MP) of artificial ventilation, the energy transferred to the respiratory system per time unit, is a chief determinant for the establishment of adequate gas exchange. Calculated MP, the product of the applied airway pressure and minute ventilation, may therefore serve as an estimate for the workload imposed on respiratory muscles when switching to spontaneous breathing, which is a crucial factor when evaluating patients’ ability to wean from mechanical ventilation. In a recent study, MP normalized to dynamic lung thorax compliance, assessed immediately before the first spontaneous breathing trial (SBT) upon admission to the weaning center, was independently associated with failure of prolonged weaning [7].

The aim of the present study was to assess the diagnostic accuracy of MP in predicting the outcome of prolonged weaning in tracheotomized patients, treated at a specialized weaning center.

## Methods

This was a prospective, observational study conducted at a national weaning center in Germany. The study was approved by the local institutional review board for human studies (Ethics Committee of the State Chamber of Physicians of Baden-Wuerttemberg, Germany, file number F-2018-116), and written informed consent was obtained from all patients or a legal representative.

### Patient selection

Patients were included in the analyses if they were referred because of evident failure to wean from tracheostomy ventilation, and if they met the criteria of prolonged weaning, classified as Category 3 as defined by Boles and colleagues [8]. Exclusion criteria were a confirmed diagnosis of neuromuscular disease, death prior to weaning completion, and declined consent for participation.

### Ventilator weaning

Weaning was systematically performed according to the recommendations of Boles and colleagues [8], and the national guidelines on prolonged weaning [9], including protocol-based increasing periods of unassisted breathing through a tracheostomy collar (weaning trials) [10]. In the intervals between SBT, all patients were ventilated in the pressure-controlled, assisted-controlled (A/C) mode (Vivo 50/55, Breas Medical AB, Moelnlycke, Sweden) to recover from the imposed work of breathing during SBT (further details can be found in the online supplement).

### Variables analyzed to predict weaning outcome

**Fig. 1:**
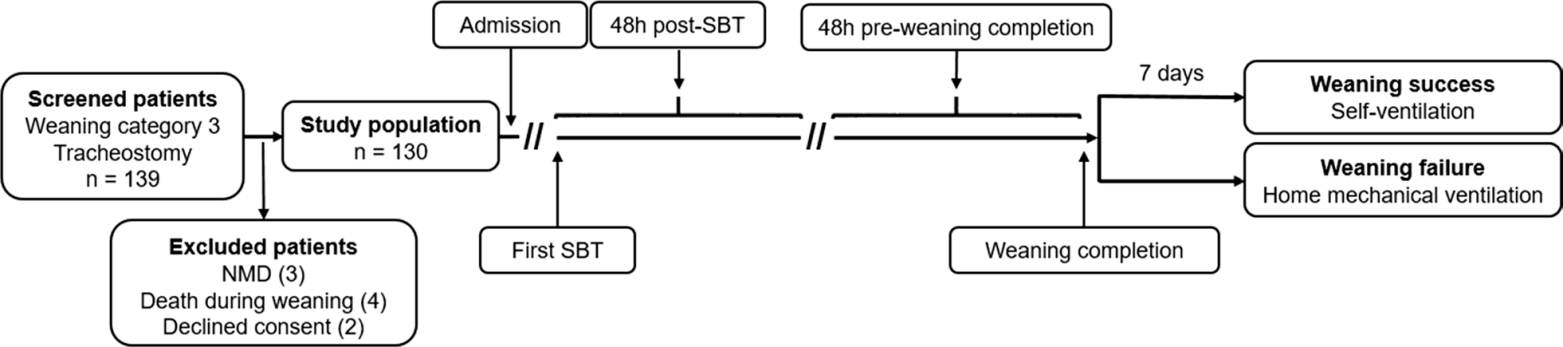
Study flow and timeline regarding the assessment of variables used to predict weaning outcome. Weaning success was defined as spontaneous breathing without signs of ventilatory failure for more than seven consecutive days after last ventilator attachment. *NMD* neuromuscular disease, *SBT* spontaneous breathing trial

Ventilator variables and the corresponding arterial blood gas analysis were collected 48 h after the first SBT upon admission to the weaning center and 48 h before weaning completion, with the median of these values used for the analyses (Fig. 1).

The following parameters were calculated from the collected variables: dynamic lung-thorax compliance (LTC_dyn_) [11], mechanical power [12, 13], and ventilatory ratio (VR) [14]. Specific MP indices were calculated by normalizing absolute values to predicted body weight (a surrogate of the total lung capacity, PBW-MP) [15], and to LTC_dyn_ (reflecting actual ventilated lung volume, LTC_dyn_-MP) [7]. Finally, LTC_dyn_-MP was corrected for the corresponding mechanical ventilation P_a_CO_2_ [7] (simulating isocapnic conditions), termed the “power index of the respiratory system” (PI_rs_), a surrogate of applied specific power necessary to provide adequate decarboxylation (further details can be found in the online supplement).

### Classification of outcomes

Patients were classified at the end of ventilator weaning into two categories: patients with weaning success, and those with weaning failure.

Successful weaning was defined as spontaneous breathing for more than seven consecutive days without concomitant clinical or laboratory signs of chronic ventilatory insufficiency after weaning completion. Weaning completion was determined by the last day on which the patient was attached to the ventilator. Weaning failure referred to a state of chronic ventilatory insufficiency in the course of weaning, defined as recurrent hypercapnia during daily weaning trials, preventing the extension of spontaneous respiration (P_a_CO_2_ > 45.0 mmHg on at least two occasions), or hypercapnia occurring during spontaneous breathing within seven consecutive days after weaning completion [7] (median P_a_CO_2_ > 45.0 mmHg derived from the highest measured P_a_CO_2_ on each of the seven days), usually requiring resumption of ventilation with transition to home mechanical ventilation (either by face mask or by tracheostomy tube).

Based on these criteria, the decision to terminate the weaning process was finally made by an interdisciplinary team including the attending physician and the respiratory therapist. Domiciliary ventilation was prescribed for at least 6 h per day [16], with the duration adjusted to patients’ need, aiming at sufficiently unloading respiratory muscles, indicated by normocapnia during both mechanical and self-ventilation.

### Sample size determination and statistical analysis

Descriptive and frequency statistics were used to summarize patients’ demographics and baseline characteristics. Differences between groups in categorical variables were analyzed using Chi-square or Fisher’s exact tests, as appropriate. Continuous variables were subjected to Kolmogorov–Smirnov normality test for homogeneity of variance, and, depending on statistical distribution, either Student’s *t*-test or Mann–Whitney *U*-test was used to examine differences in these parameters.

To assess the ability of the variables to predict weaning outcome, we performed a receiver operating characteristic (ROC) curve analysis in the entire study population with diagnostic performance expressed as area under the ROC curve (AUROC). We also performed a prospective, stratified, 2-times repeated, twofold cross-validation [17] (see Additional file 1: Figure S1, Figure S2, Table S1), with the resulting performance of each index derived from the test sets expressed as sensitivity, specificity, positive/negative predictive value, accuracy, positive/negative likelihood ratio, diagnostic odds ratio (DOR), F_1_ score, and Matthews correlation coefficient (MCC).

Pearson’s *r* was used for assessment of correlation between the index tests and daily duration of mechanical ventilation following (unsuccessful) weaning completion.

Based on an α-level (type-I error) of 0.05 with power (1‒β) of 80% and prevalence of weaning failure (as defined above) of 40% [7], we calculated a sample size of 124 patients to capture variables with at least moderate diagnostic accuracy (AUROC > 0.70) from the training sets. All tests were two-tailed; statistical significance was indicated by *p* < 0.05. All analyses were performed using MedCalc statistical software version 19.2.5 (MedCalc Software Ltd, Ostend, Belgium). Further details on the statistical methods can be found in the online supplement.

## Results

Of 139 consecutive patients screened for eligibility between March 2019 and August 2020, 130 (93.5%) were included in the analyses. Reasons for exclusion were a confirmed diagnosis of neuromuscular disease in three patients, four patients died during weaning, and two patients declined consent.

Clinical characteristics differed between patients with successful and unsuccessful weaning mainly in terms of smoking history, presence of chronic obstructive pulmonary disease (COPD) as a comorbidity, and acute exacerbation of COPD as the reason for respiratory failure (Table 1).

**Table 1:** Clinical characteristics on admission to the weaning center— comparison of patients with weaning success and weaning failure

Clinical characteristics	All patients (n = 130)	Weaning success (n = 86)	Weaning failure (n = 44)	*P* value^a^
Age (years)	69 (60–76)	68 (59–76)	70 (63–75)	0.609^c^
Gender (male)	82 (63.1)	59 (68.8)	23 (52.3)	0.069^d^
Body mass index (kg/m^2^)	27.9 (± 6.9)	27.3 (± 6.6)	29.1 (± 7.4)	0.369^c^
Obesity (BMI ≥ 30 kg/m^2^)	40 (30.8)	24 (27.9)	16 (36.4)	0.325^d^
Smoking history	48 (36.9)	21 (24.5)	27 (61.4)	** < 0.001**^**d**^
APACHE-II (points)	15.9 (± 5.3)	15.8 (± 5.3)	15.9 (± 5.4)	0.905^b^
Albumin (g/dL)	2.2 (± 0.5)	2.2 (± 0.6)	2.2 (± 0.5)	0.338^c^
Ventilator days on admission	25 (16–34)	27 (17–35)	23 (16–30)	0.123^c^
Time from Intubation to tracheostomy (days)	12 (7–18)	13 (7–19)	10 (6–15)	0.057^c^
ECLA	14 (10.8)	13 (15.1)	1 (2.3)	**0.026**^**d**^
*Reason for mechanical ventilation*				
Pneumonia	51 (39.2)	31 (36.0)	20 (45.5)	0.300^d^
SARS-CoV-2 infection	4 (3.1)	4 (4.7)	0 (0.0)	–
Surgery	32 (24.6)	25 (29.1)	7 (15.9)	0.101^d^
Cardiopulmonary resuscitation	10 (7.7)	8 (9.3)	2 (4.5)	0.337^d^
Acute exacerbation of COPD	10 (7.7)	2 (2.3)	8 (18.2)	**0.001**^**d**^
Acute heart failure	6 (4.6)	5 (5.8)	1 (2.3)	0.663^e^
Sepsis (including septic shock)	7 (5.4)	5 (5.8)	2 (4.5)	1.000^e^
Other	17 (13.1)	10 (11.6)	7 (15.9)	0.495^d^
*Comorbidities*				
Charlson comorbidity index (points)	5.5 (± 2.3)	5.4 (± 2.4)	5.6 (± 2.1)	0.611^c^
Renal insufficiency	46 (35.4)	36 (41.9)	10 (22.7)	**0.032**^**d**^
Hemodialysis	24 (18.5)	21 (24.4)	3 (6.8)	0.289^d^
Diabetes mellitus	35 (26.9)	20 (23.3)	15 (34.1)	0.189^d^
Coronary artery disease	33 (25.4)	23 (26.7)	10 (22.7)	0.620^d^
COPD	30 (23.1)	5 (5.8)	25 (56.8)	** < 0.001**^**d**^
Chronic heart failure	17 (13.1)	14 (16.3)	3 (6.8)	0.132^d^
Malignancy	10 (7.7)	7 (8.1)	3 (6.8)	0.790^d^
Hepatopathy	7 (5.4)	5 (5.8)	2 (4.5)	1.000^e^
Interstitial lung disease	8 (6.2)	6 (7.0)	2 (4.5)	0.716^e^

Continuous variables are presented as arithmetic means (± standard deviation) or median (– interquartile range [IQR]); categorical variables are presented as number (%)

*BMI* body mass index, *APACHE-II* Acute Physiology and Chronic Health Evaluation II score, *ECLA* extracorporeal lung assistance (in acute respiratory failure), *SARS-CoV-2* severe acute respiratory syndrome-Coronavirus 2, *COPD* chronic obstructive pulmonary disease

^a^*P* value for differences between patients with weaning success and weaning failure

^b^Student’s *t*-test

^c^Mann–Whitney *U*-test

^d^Chi-squared test

^e^Fisher’s exact test

Weaning failure occurred in 44 patients (33.8%), 42 of whom remained ventilated on hospital discharge (19 on non-invasive home mechanical ventilation and 23 discharged with permanent tracheostomy ventilation) (see Additional file 1: Table S2).

Patients’ baseline clinical characteristics and outcomes were equally distributed after each randomization to one of the two groups (see Additional file 1: Table S1). Table 2 shows the mean threshold values derived from the training sets that best predicted weaning failure.

**Table 2:** Threshold values of variables used to predict weaning outcome—mean values derived from the training sets

Variables	Failure of prolonged weaning	
	Post-SBT	Pre-weaning completion
P_a_CO_2_ on MV (mmHg)	> 35.3	> 34.6
VR	> 1.23	> 1.23
LTC_dyn_ (mL/cmH_2_O)	≤ 31.2	≤ 33.6
MP (J/min)	> 20.8	> 20.3
PBW-MP (J/min/kg)	> 0.3164	> 0.3158
LTC_dyn_-MP (cmH_2_O^2^/min)	> 6496	> 6341
PI_rs_^1.0^ (cmH_2_O^2^/min)	> 5020	> 4915
PI_rs_^2.0^ (cmH_2_O^2^/min)	> 3895	> 3704

> / ≤ indicate whether values above/below the threshold value predicted failure of prolonged weaning. The associated criterion is the threshold value that minimized the difference between sensitivity and specificity of the test, graphically corresponding to the intersection of the line connecting the left-upper corner and the right-lower corner of the unit square and the ROC curve

*SBT* spontaneous breathing trial, *MV* mechanical ventilation, *VR* ventilatory ratio, *LTC*_*dyn*_ dynamic lung-thorax compliance, *MP* mechanical power, *PBW-MP* mechanical power normalized to predicted body weight, *LTC*_*dyn*_*-MP* mechanical power normalized to dynamic lung-thorax compliance, *PI*_*rs*_ power index of the respiratory system

A good diagnostic performance in predicting failure of prolonged weaning from values collected 48 h after the first SBT was shown for P_a_CO_2_ on mechanical ventilation (DOR 11.1, MCC 0.50, AUROC 0.81 [0.73‒0.87]), and the PI_rs_ (DOR 15.3, MCC 0.52, AUROC 0.86 [0.79‒0.92]) (Tables 3 and 4).

**Table 3:** Cross-validated performance of variables analyzed to predict weaning outcome—mean values derived from the test sets

Variables	Failure of prolonged weaning									
	Sens	Spec	PPV	NPV	Accuracy	PLR	NLR	DOR	F_1_	MCC
*Post-SBT*										
P_a_CO_2_ on MV	78 (56–93)	73 (58–85)	61 (47–73)	87 (75–94)	75 (63–85)	3.1 (1.7–5.5)	0.3 (0.7–0.1)	11.1	0.68	0.50
VR	73 (50–89)	71 (55–84)	56 (43–69)	84 (72–91)	72 (59–82)	2.6 (1.5–4.4)	0.4 (0.8–0.2)	7.2	0.63	0.42
LTC_dyn_	68 (46–85)	71 (55–84)	54 (40–67)	82 (71–89)	70 (57–81)	2.4 (1.4–4.1)	0.4 (0.8–0.3)	7.7	0.60	0.38
MP	66 (44–84)	64 (48–78)	48 (36–53)	79 (67–87)	65 (52–76)	1.8 (1.1–3.0)	0.5 (1.0–0.3)	4.5	0.55	0.28
PBW-MP	68 (45–86)	68 (52–81)	53 (40–66)	81 (69–89)	68 (55–79)	2.3 (1.3–4.1)	0.5 (0.9–0.3)	5.6	0.59	0.35
LTC_dyn_-MP	73 (51–88)	67 (54–80)	53 (41–65)	83 (71–90)	69 (56–80)	2.3 (1.4–3.9)	0.4 (0.8–0.2)	7.3	0.61	0.38
PI_rs_^1.0^	74 (52–89)	73 (58–86)	58 (45–71)	85 (73–92)	73 (61–84)	2.9 (1.6–4.1)	0.4 (0.7–0.2)	11.3	0.65	0.45
PI_rs_^2.0^	78 (56–92)	76 (61–88)	63 (49–75)	87 (75–94)	77 (65–86)	3.4 (1.9–6.2)	0.3 (0.6–0.1)	15.3	0.70	0.52
*Pre-weaning completion*										
P_a_CO_2_ on MV	77 (55–92)	76 (60–87)	62 (48–74)	87 (75–93)	76 (64–86)	3.2 (1.8–5.8)	0.3 (0.7–0.1)	11.6	0.69	0.51
VR	70 (47–88)	66 (50–79)	52 (39–64)	81 (69–90)	67 (55–78)	2.1 (1.3–3.5)	0.5 (0.9–0.2)	4.8	0.59	0.35
LTC_dyn_	69 (44–88)	76 (61–87)	56 (41–69)	86 (75–92)	74 (62–84)	3.2 (1.7–6.3)	0.4 (0.8–0.2)	9.4	0.61	0.43
MP	70 (48–87)	60 (44–74)	48 (37–59)	80 (67–89)	63 (51–75)	1.8 (1.1–2.9)	0.5 (1.0–0.2)	3.8	0.57	0.29
PBW-MP	68 (45–86)	67 (51–80)	51 (39–64)	81 (68–89)	67 (55–78)	2.1 (1.2–3.5)	0.5 (0.9–0.3)	4.6	0.58	0.33
LTC_dyn_-MP	74 (53–88)	77 (62–89)	62 (47–75)	86 (75–92)	76 (64–86)	3.3 (1.8–6.2)	0.3 (0.7–0.2)	18.5	0.67	0.49
PI_rs_^1.0^	82 (61–94)	81 (66–91)	68 (53–81)	90 (78–95)	81 (70–90)	4.4 (2.3–8.5)	0.2 (0.6–0.1)	42.9	0.74	0.61
PI_rs_^2.0^	88 (67–97)	83 (69–93)	73 (58–84)	93 (81–97)	85 (74–92)	5.9 (2.8–13)	0.2 (0.5–0.1)	52.8	0.79	0.68

Assessment of mean sensitivity and specificity, positive and negative predictive value, positive and negative likelihood ratio, diagnostic odds ratio, F_1_ score, and Matthews correlation coefficient (with 95% confidence intervals)

*SBT* spontaneous breathing trial, *Sens* sensitivity, *Spec* specificity, *PPV* positive predictive value, *NPV* negative predictive value, *PLR* positive likelihood ratio, *NLR* negative likelihood ratio, *DOR* diagnostic odds ratio, *F*_*1*_ F_1_ score, *MCC* Matthews correlation coefficient, *MV* mechanical ventilation, *VR* ventilatory ratio, *LTC*_*dyn*_ dynamic lung-thorax compliance, *MP* mechanical power, *PBW-MP* mechanical power normalized to predicted body weight, *LTC*_*dyn*_*-MP* mechanical power normalized to dynamic lung-thorax compliance, *PI*_*rs*_ power index of the respiratory system

**Table 4:** Area under the ROC curve for each variable—all patients

Variables	Failure of prolonged weaning	
	Post-SBT	Pre-weaning completion
P_a_CO_2_ on MV	0.81 (0.73–0.87)	0.86 (0.78–0.91)
VR	0.78 (0.70–0.85)	0.74 (0.66–0.81)
LTC_dyn_	0.74 (0.65–0.81)	0.82 (0.75–0.89)
MP	0.74 (0.65–0.81)	0.70 (0.61–0.77)
PBW-MP	0.76 (0.68–0.83)	0.75 (0.66–0.82)
LTC_dyn_-MP	0.79 (0.71–0.86)	0.86 (0.78–0.91)
PI_rs_^1.0^	0.84 (0.76–0.90)	0.91 (0.84–0.95)
PI_rs_^2.0^	0.86 (0.79–0.92)	0.92 (0.86–0.96)

The accuracy of each variable in the whole study population presented as the area under the ROC curve with 95% confidence intervals

*SBT* spontaneous breathing trial, *MV* mechanical ventilation, *VR* ventilatory ratio, *LTC*_*dyn*_ dynamic lung-thorax compliance, *MP* mechanical power, *PBW-MP* mechanical power normalized to predicted body weight, *LTC*_*dyn*_*-MP* mechanical power normalized to dynamic lung-thorax compliance, *PI*_*rs*_ power index of the respiratory system

**Fig. 2:**
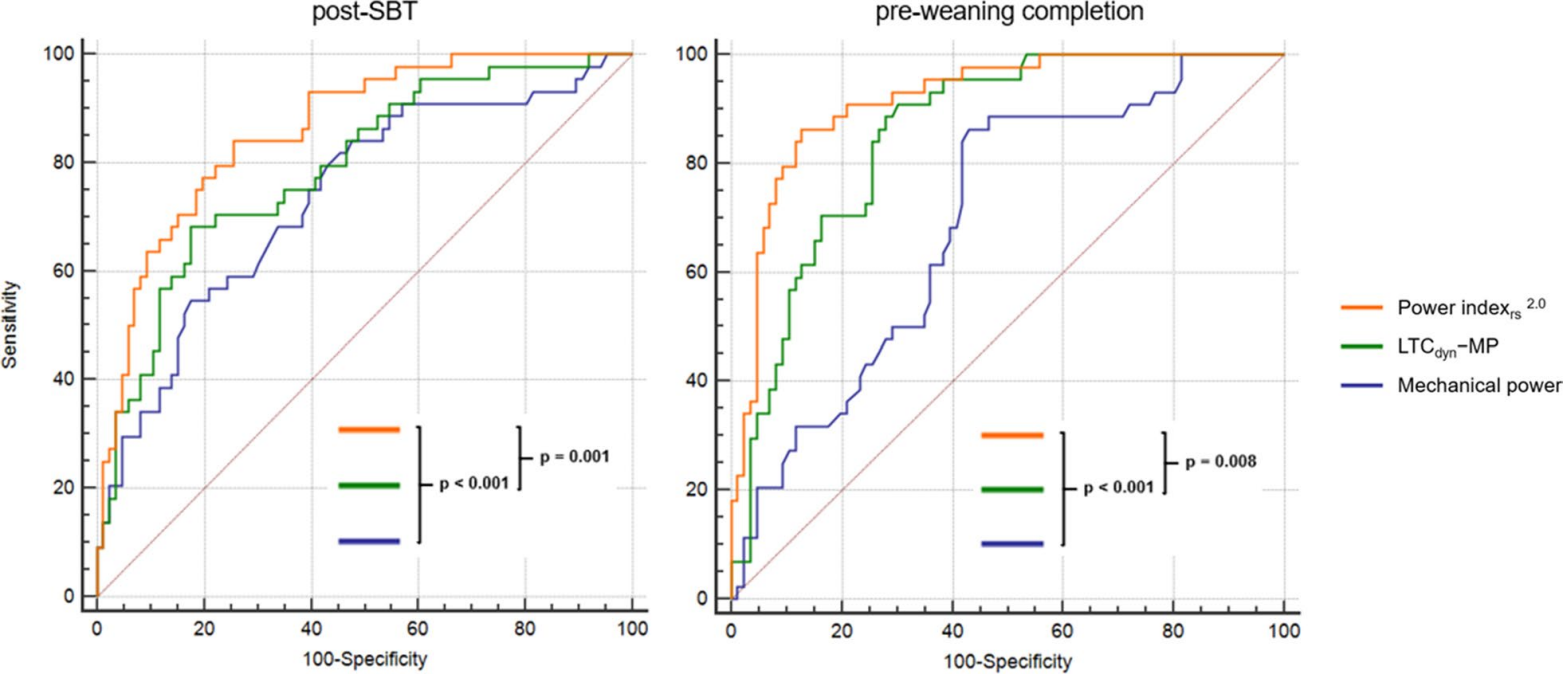
Comparison of ROC curves for mechanical power, LTC_dyn_-MP, and the power index_rs_^2.0^ predicting the outcome of prolonged weaning—all patients. *ROC* receiver operating characteristic curve, *LTC*_*dyn*_*-MP* mechanical power normalized to dynamic lung-thorax compliance

An excellent performance in predicting unsuccessful weaning from values assessed 48 h before weaning completion was observed for LTC_dyn_-MP (DOR 18.5, MCC 0.49, AUROC 0.86 [0.78‒0.91]) and especially for PI_rs_ (sensitivity 88%, specificity 83%, DOR 52.8, MCC 0.68, AUROC 0.92 [0.86‒0.96]). In contrast, absolute mechanical power had moderate diagnostic accuracy for both timepoints, whereas PI_rs_ predicted weaning failure significantly better than MP or LTC_dyn_-MP (Fig. 2).

In the 62 patients with median PI_rs_^2.0^ < 3704 cmH_2_O^2^/min (corresponding to the mean threshold 48 h before weaning completion derived from the training sets, Table 2) at both the beginning and the end of weaning, only three patients (4.8%) remained ventilator-dependent on discharge.

Since the diagnostic accuracy of an index derived from cross-validation largely depends on the selected threshold, a second analysis was performed using the criteria associated with the Youden index, resulting in similar diagnostic performance of variables derived from mechanical power (see Additional file 1: Table S3).

When comparing the discriminatory performance of PBW-MP and LTC_dyn_-MP, both of which are indices relating transferred power to surrogates of lung volume, LTC_dyn_-MP consistently performed better in predicting weaning outcome (Table 4). These differences in AUROC reached statistical significance 48 h before weaning completion (0.75 [0.66‒0.82] vs 0.86 [0.78‒0.91], p = 0.004).

Comparison of the diagnostic performance of PI_rs_^2.0^ from patients with less and more than 50% of variables recorded during pressure-assisted ventilation revealed neither a significant difference in predicting weaning outcome from values collected 48 h after the first SBT (AUROC 0.92 [0.83‒0.98] vs 0.80 [0.69‒0.89], p = 0.056), nor from values assessed 48 h before weaning completion (0.95 [0.85‒0.99] vs 0.91 [0.83‒0.96], p = 0.423).

Finally, Pearson’s *r* showed a significant correlation of LTC_dyn_-MP (*r* = 0.44, 95%CI 0.29‒0.57; p < 0.001) and PI_rs_ (0.61 [0.49‒0.71]; p < 0.001) at end of weaning with the duration of daily home ventilation (12.0 ± 7.9 h in weaning failure patients).

Further details on differences in ventilator variables and mechanical power indices between the successful and unsuccessful weaning group (Additional file 1: Table S4), and ROC curves of selected variables (Additional file 1: Figure S3) can be found in the online data supplement (see Additional file 1).

## Discussion

The aim of the present study was to determine the diagnostic accuracy of MP in predicting successful weaning from prolonged tracheostomy ventilation. Absolute MP showed poor discriminatory performance, whereas specific MP (MP normalized to surrogates of lung dimension, including well-inflated lung tissue determined by computed tomography analysis [18], functional residual capacity, PBW or LTC_dyn_) had good diagnostic accuracy, further improved by correction for the corresponding P_a_CO_2_ on mechanical ventilation. Moreover, discriminatory performance of the MP indices increased over the course of weaning, with maximum accuracy observed immediately before weaning completion.

The concept of MP is relatively new and has been studied mainly in the context of acute respiratory distress syndrome, since calculated MP includes all ventilator-related variables (e.g. respiratory rate, tidal volume, inspiratory pressure) that, if inexpertly applied, may result in ventilator-induced lung injury [19]. In a previous observational study in prolonged mechanically ventilated, tracheotomized patients, LTC_dyn_-MP was independently associated with weaning outcome [7], most probably because it agrees well with the pathophysiological basis of prolonged weaning, which is mainly perceived as an imbalance between load (equal to the MP generated by respiratory muscles necessary to provide adequate gas exchange) and capacity of the respiratory pump [5‒6].

Specific MP, given it considers lung volume, adjusts for age, gender, and body height [20]. Furthermore, LTC_dyn_-MP accounts for changes in airway resistance and chest wall elastance, both of which substantially contribute to the total energy required to inflate the lungs. From a physical perspective, LTC_dyn_-MP represents a pressure–time quotient (referred to as “stress intensity”) describing the relationship between respiratory rate, pressure and power. Increasing respiratory rate leads to a linear rise in energy transfer, while an increase in pressure (concomitantly increasing tidal volume) results in an exponential increment in power [19].

The formula also reveals the effects of a change in PEEP on the applied specific power in the pressure-controlled ventilation mode. Provided all other variables remain constant, an increase in PEEP (accompanied by a decrease in driving pressure) results in a reduction in the specific power. Even if an increase in PEEP would lead to recruitment of lung tissue (accompanied by an increase in tidal volume), absolute MP might increase but specific power remains constant, since ventilated lung volume increases to the same extent (reflected by the change in respiratory system compliance). If PEEP reaches the preset inspiratory pressure (driving pressure equals zero), there is no more power transfer from the ventilator to the respiratory system.

The PI_rs_ further normalizes applied specific MP to the associated P_a_CO_2_ (a surrogate for alveolar ventilation), simulating isocapnic conditions and thereby resulting in improved accuracy in predicting the outcome of prolonged weaning. However, this only approximates respiratory physiology, since an increase in pressure (despite increasing tidal volume) will probably not decrease P_a_CO_2_ when applying an extremely high inspiratory pressure above the upper inflection point of the respiratory system pressure–volume curve, resulting in increased dead space ventilation due to regional lung over-distension [21]. Moreover, PI_rs_ does not account for another phenomenon called pendelluft, which may occur during assisted ventilation with low pressure support, leading to increased inspiratory effort with pendular movement of gas between different lung regions, thereby decreasing CO_2_ removal [22]. Nevertheless, our results suggest that PI_rs_ is sufficiently accurate in estimating the MP generated either by the ventilator or by respiratory muscles necessary to provide adequate gas exchange, with the observed increase in diagnostic accuracy of MP indices over the course of weaning most probably explained by our therapeutic interventions, which aimed at improving respiratory mechanics and pulmonary gas exchange, leading to a fall of respiratory muscle workload below the individual fatigue threshold in successfully weaned patients. The observed correlation between specific MP and daily duration of prescribed domiciliary ventilation, which was necessary to sufficiently unload the respiratory pump, further indicates the utility of these indices in estimating respiratory muscle workload during self-ventilation.

Several predictors of weaning and extubation outcome have previously displayed substantial differences in diagnostic accuracy in short-term mechanically ventilated patients [4]. In general, these parameters are determined after a short period of spontaneous breathing (a few minutes after the beginning or at the end of a weaning trial) with or without minimal ventilator assistance. Several of these parameters showed only poor to moderate diagnostic performance in predicting weaning outcome (e.g. maximum inspiratory pressure [23], respiratory rate, tidal volume, or oxygenation [24]). Several attempts have been made to increase accuracy by combining individual parameters into integrative indices. Examples are the CROP [23, 24], CORE [26], Weaning [27], and Integrative weaning [28] indices. CROP and CORE unify respiratory system compliance, (maximum) inspiratory pressure, tidal volume, oxygenation, and respiratory rate, thereby significantly increasing discriminatory performance in predicting the outcome of a weaning trial [25, 26]. However, assessment of some of these variables is complex (depending on patient’s respiratory drive), and none of these indices account for the exponential relationship between pressure and power, and none have been validated in prolonged mechanically ventilated patients. In contrast, mechanical power indices (LTC_dyn_-MP and PI_rs_) derive from a physical equation, have a sound pathophysiological rationale, and can be easily assessed during mechanical ventilation without disconnecting the patient from the ventilator.

PI_rs_ approximates the load imposed on respiratory muscles during spontaneous breathing. However, since development of hypercapnic respiratory failure is determined by an imbalance between work of breathing and respiratory pump capacity [5, 6], one also has to consider the latter when judging a patient’s ability to wean from prolonged ventilation. Several studies have assessed trajectories of respiratory muscle function in patients in intensive care, and the impact of this trajectory on mortality and ventilator weaning. While development of, and risk factors for, ventilator-induced diaphragmatic weakness are well described [29‒31], little is known about potential reversibility. In a physiological study, Grassi and co-workers demonstrated a correlation between mode of ventilation and changes in diaphragmatic thickness [32]. After early thinning during a short period of controlled ventilation, thickness and thickening fraction recuperated in about 50% of patients within one week after switching to assisted ventilation. Moreover, Jubran and colleagues showed that respiratory strength (assessed by maximum inspiratory pressure) was well maintained in prolonged mechanically ventilated, tracheotomized patients, and did not significantly change between hospital admission and discharge. They concluded that an increase in muscle strength was not the major determinant of ventilator detachment, but that reduced strength may be a factor in weaning failure [1]. Both studies indicate that in most patients respiratory muscle strength recovers early in the course of weaning (e.g. from the moment of switching to assisted ventilation), with weaning outcome mainly determined by the load imposed during spontaneous breathing, consistent with the present results. Nevertheless, prevalence and magnitude of respiratory muscle weakness related to ventilator-associated diaphragmatic dysfunction, bilateral phrenic nerve injury, or pre-existing neuromuscular disease significantly impact the individual fatigue threshold above which ventilatory failure occurs, consequently determining the diagnostic accuracy of MP indices in predicting weaning outcome.

Our study has several strengths. The broad range of medical and surgical patients included increases the applicability of the results. Threshold values and their (internal) validation were assessed prospectively, with the cross-validation technique providing a more thorough estimation of diagnostic performance and generalizability by reducing selection bias (compared to allocating patients to groups based on the time they entered the study). Both the index tests and the reference standard (P_a_CO_2_ during spontaneous breathing) are objective criteria, further reducing the likelihood of bias when determining diagnostic accuracy in predicting weaning outcome. However, this study also has some limitations. Since this was a single-center analysis, external validity is uncertain, despite the broad range of patients we recruited. In addition, MP was calculated using a surrogate formula, proposed for pressure-controlled ventilation [12]. However, in a recent study this simplified, surrogate formula, working under the assumption of a perfectly squared delivered pressure wave, performed better than a more comprehensive formula, without the need for complex calculations [13]. Furthermore, 50.5% of all variables analyzed were recorded during assisted-ventilation. Since in a single patient the same inspiratory pressure and PEEP was applied during both a controlled and an assisted breath (in the assisted-controlled mode), leading to identical transrespiratory pressures, absolute MP simply depended on the resulting minute ventilation. In contrast, at a given inspiratory and expiratory pressure, LTC_dyn_-MP does not account for changes in minute ventilation when increasing tidal volume due to activation of the diaphragm on assisted-ventilation, potentially leading to a distortion of P_a_CO_2_ and consequently PI_rs_.

## Conclusions

In conclusion, mechanical power normalized to dynamic lung thorax compliance, a surrogate for the applied power per unit of ventilated lung volume, may serve as an estimate of the workload imposed on respiratory muscles during spontaneous breathing, thereby predicting patients’ ability to wean from prolonged tracheostomy ventilation. Future changes in clinical practice of tracheostomy weaning could involve weaning protocols including this new predictor in order to help clinicians in guiding the weaning process (e.g. to control the degree of daily extension of spontaneous breathing), with the aim of reducing weaning duration. Further studies are needed to confirm our results.

## Supplementary Information

**Additional file 1**: Figure S1, Statistical methods—ROC curve analysis and prospective 2-times repeated, twofold cross validation; Figure S2, 2 × 2 confusion matrix—Metrics of diagnostic accuracy; Table S1, Clinical characteristics on admission to the weaning center—comparison of groups A/B and C/D; Table S2, Results of prolonged weaning—comparison of patients with weaning success and weaning failure; Table S3, Cross-validated performance of variables derived from the mechanical power analyzed to predict weaning outcome—mean values derived from the test sets; Table S4, Ventilator variables and mechanical power indices predicting the outcome of prolonged weaning—comparison of patients with weaning success and weaning failure; Figure S3, ROC curves for selected variables and mechanical power indices derived from the whole study population 48 h before weaning completion (PDF 1.0 MB).

## Data Availability

The datasets used and/or analyzed during the current study are available from the corresponding author on reasonable request.

## Abbreviations

aBGA: Arterial blood gas analysis
ACC: Accuracy
APACHE-II: Acute Physiology and Chronic Health Evaluation II score
AUROC: Area under the receiver operating characteristic curve
BMI: Body mass index
COPD: Chronic obstructive pulmonary disease
DOR: Diagnostic odds ratio
ECLA: Extracorporeal lung assistance
F_1_: F_1_ score
F_i_O_2_: Fraction of inspired oxygen
HMV-IMV: Home mechanical ventilation‒invasive mechanical ventilation
HMV-NIV: Home mechanical ventilation‒non-invasive mechanical ventilation
IMV: Invasive mechanical ventilation
LOS: Length of stay
LTC_dyn_: Dynamic lung-thorax compliance
LTC_dyn_-MP: MP normalized to dynamic lung-thorax compliance
LTOT: Long-term oxygen therapy
MCC: Matthews correlation coefficient
MP: Mechanical power
MV: Mechanical ventilation
NLR: Negative likelihood ratio
No.: Number
NPV: Negative predictive value
PBW-MP: MP normalized to predicted body weight
PI_rs_: Power index of the respiratory system
PLR: Positive likelihood ratio
PPV: Positive predictive value
ROC: Receiver operating characteristic (curve)
SB: Spontaneous breathing
SBT: Spontaneous breathing trial
Sens: Sensitivity
Spec: Specificity
VR: Ventilatory ratio

## References

[1] Jubran A, Grant BJB, Duffner LA (2019). Long-term outcome after prolonged mechanical ventilation. Am J Respir Crit Care Med.

[2] Bonnici DM, Sanctuary T, Warren A (2016). Prospective observational cohort study of patients with weaning failure admitted to a specialist weaning, rehabilitation and home mechanical ventilation centre. BMJ Open.

[3] Ghiani A, Sainis A, Sainis G, Neurohr C (2019). Anemia and red blood cell transfusion practice in prolonged mechanically ventilated patients admitted to a specialized weaning center: an observational study. BMC Pulm Med.

[4] Baptistella AF, Sarmento FJ, Ribeiro da Silva K (2018). Predictive factors of weaning from mechanical ventilation and extubation outcome: a systematic review. J Crit Care.

[5] Purro A, Appendini L, De Gaetano A, Gudjonsdottir M, Donner CF, Rossi A (2000). Physiologic determinants of ventilator dependence in long-term mechanically ventilated patients. Am J Respir Crit Care Med.

[6] Carlucci A, Ceriana P, Prinianakis G, Fanfulla F, Colombo R, Nava S (2009). Determinants of weaning success in patients with prolonged mechanical ventilation. Crit Care.

[7] Ghiani A, Paderewska J, Sainis A, Crispin A, Walcher S, Neurohr C (2020). Variables predicting weaning outcome in prolonged mechanically ventilated tracheotomized patients: a retrospective study. J Intensive Care.

[8] Boles JM, Bion J, Connors A (2007). Weaning from mechanical ventilation. Eur Respir J.

[9] Schönhofer B, Geiseler J, Dellweg D (2019). Prolonged weaning: S2k-guideline published by the German Respiratory Society. Pneumologie.

[10] Jubran A, Grant BJ, Duffner LA (2013). Effect of pressure support vs unassisted breathing through a tracheostomy collar on weaning duration in patients requiring prolonged mechanical ventilation: a randomized trial. JAMA.

[11] Okabe Y, Asaga T, Bekku S (2018). Lung-thorax compliance measured during a spontaneous breathing trial is a good index of extubation failure in the surgical intensive care unit: a retrospective cohort study. J Intensive Care.

[12] Becher T, van der Staay M, Schädler D, Frerichs I, Weiler N (2019). Calculation of mechanical power for pressure-controlled ventilation. Intensive Care Med.

[13] Chiumello D, Gotti M, Guanziroli M (2020). Bedside calculation of mechanical power during volume- and pressure controlled mechanical ventilation. Crit Care.

[14] Sinha P, Fauvel NJ, Singh P, Soni N (2013). Analysis of ventilatory ratio as a novel method to monitor ventilatory adequacy at the bedside. Crit Care.

[15] Brower RG, Matthay MA, Morris A, Schoenfeld D, Thompson BT, Wheeler A (2000). Ventilation with lower tidal volumes as compared with traditional tidal volumes for acute lung injury and the acute respiratory distress syndrome. N Engl J Med.

[16] Windisch W, Dreher M, Geiseler J (2017). Guidelines for non-invasive and invasive home mechanical ventilation for treatment of chronic respiratory failure: update 2017. Pneumologie.

[17] Arlot S, Celisse A (2010). A survey of cross-validation procedures for model selection. Stat Surv.

[18] Coppola S, Caccioppola A, Froio S (2020). Effect of mechanical power on intensive care mortality in ARDS patients. Crit Care.

[19] Gattinoni L, Tonetti T, Cressoni M (2016). Ventilator-related causes of lung injury: the mechanical power. Intensive Care Med.

[20] Quanjer PH, Stanojevic S, Cole TJ (2012). Multi-ethnic reference values for spirometry for the 3–95-yr age range: the global lung function 2012 equations. Eur Respir J.

[21] Blanch L, Lopez-Aguilar J, Lucangelo U (2016). Dead space in acute respiratory distress syndrome: more than a feeling!. Crit Care.

[22] Coppadoro A, Grassi A, Giovannoni C (2020). Occurrence of pendelluft under pressure support ventilation in patients who failed a spontaneous breathing trial: an observational study. Ann Intensive Care.

[23] Wu YK, Kao KC, Hsu KH, Hsieh MJ, Tsai YH (2009). Predictors of successful weaning from prolonged mechanical ventilation in Taiwan. Respir Med.

[24] Savi A, Teixeira C, Silva JM (2012). Weaning predictors do not predict extubation failure in simple-to-wean patients. J Crit Care.

[25] Yang KL, Tobin MJ (1991). A prospective study of indexes predicting the outcome of trials of weaning from mechanical ventilation. N Engl J Med.

[26] Delisle S, Francoeur M, Albert M, Ouellet P, Bellemare P, Arsenault P (2011). Preliminary evaluation of a new index to predict the outcome of a spontaneous breathing trial. Respir Care.

[27] Huaringa AJ, Wang A, Haro MH, Leyva FJ (2012). The weaning index as a predictor of weaning success. J Intensive Care Med.

[28] Nemer SN, Barbas CSV, Caldeira JB (2009). A new integrative weaning index of discontinuation from mechanical ventilation. Crit Care.

[29] Levine S, Nguyen T, Taylor N (2008). Rapid disuse atrophy of diaphragm fibers in mechanically ventilated humans. N Eng J Med.

[30] Dres M, Dube BP, Mayaux J (2017). Coexistance and impact of limb muscle and diaphragm weakness at time of liberation from mechanical ventilation in medical intensive care unit patients. Am J Respir Crit Care Med.

[31] Goligher EC, Dres M, Fan E (2018). Mechanical ventilation-induced diaphragm atrophy strongly impacts clinical outcomes. Am J Respir Crit Care Med.

[32] Grassi A, Ferlicca D, Lupieri E (2020). Assisted mechanical ventilation promotes recovery of diaphragmatic thickness in critically ill patients: a prospective observational study. Crit Care.

