# Supplementary material for: Mechanical power normalized to lung-thorax compliance predicts prolonged ventilation weaning failure: a prospective study
Source: BMC Pulm Med. 2021 Jun 17;21:202. doi: 10.1186/s12890-021-01566-8 (PMC8210735; doi:10.1186/s12890-021-01566-8)
Supplement: Supplementary file 1 — Additional file 1: Figure S1, Statistical methods—ROC curve analysis and prospective 2-times repeated, twofold cross validation; Figure S2, 2 × 2 confusion matrix—Metrics of diagnostic accuracy; Table S1, Clinical characteristics on admission to the weaning center—comparison of groups A/B and C/D; Table S2, Results of prolonged weaning—comparison of patients with weaning success and weaning failure; Table S3, Cross-validated performance of variables derived from the mechanical power analyzed to predict weaning outcome—mean values derived from the test sets; Table S4, Ventilator variables and mechanical power indices predicting the outcome of prolonged weaning—comparison of patients with weaning success and weaning failure; Figure S3, ROC curves for selected variables and mechanical power indices derived from the whole study population 48 h before weaning completion (PDF 1.0 MB). [file 12890_2021_1566_MOESM1_ESM.pdf]

Mechanical power normalized to lung-thorax compliance predicts prolonged ventilation weaning failure: a prospective study

Alessandro Ghiani, MD; Joanna Paderewska, MD; Swenja Walcher, RT;

and Claus Neurohr, MD

Additional file 1

1. Definitions of ventilator variables and mechanical power indices (Page 2)
2. Ventilator weaning (Page 4)
3. Statistical analysis – Metrics of diagnostic accuracy (Page 5)
4. Figure S1 (Page 6)
5. Figure S2 (Page 7)
6. Table S1 (Page 8)
7. Table S2 (Page 9)
8. Table S3 (Page 10)
9. Table S4 (Page 11)
10. Figure S3 (Page 12)
11. Abbreviation list (Page 13)
12. References (Page 15)

Definitions of ventilator variables and mechanical power indices

**Ventilatory ratio (VR)**	Ventilatory ratio (VR) is a surrogate of pulmonary dead space fraction and a simple bedside index of impaired efficiency of ventilation [1-2]:<br><br>$$VR = VE_{measured} * P_aCO_{2\text{-}measured} / VE_{predicted} * P_aCO_{2\text{-}ideal}$$<br><br>$VE_{measured}$ is the measured minute ventilation (mL/min), $P_aCO_{2\text{-}measured}$ is the measured arterial pressure of carbon dioxide (mmHg), $VE_{predicted}$ is the predicted minute ventilation calculated as predicted body weight x 1000 (mL/min), and P_aCO2_{ideal} is the expected arterial pressure of carbon dioxide in normal lungs if ventilated with the predicted minute ventilation. $P_aCO_{2\text{-}ideal}$ is set at 37.5 mmHg (5 kPa) for all patients. VR is a unitless ratio, and a value approximating 1 would represent normal ventilating lungs.
**PEEP**	Positive end-expiratory pressure (PEEP)
**IPAP**	Inspiratory positive airway pressure (IPAP) including PEEP
**Driving pressure (DP)**	Driving pressure (DP) was calculated using IPAP and PEEP:<br><br>$$DP\ (cmH_2O) = IPAP – PEEP$$
**Dynamic lung-thorax compliance (LTC_{dyn})**	Dynamic lung-thorax compliance (LTC_{dyn}) was calculated using tidal volume (VT) and driving pressure:<br><br>$$LTC_{dyn}\ (mL/cmH_2O) = VT / DP$$
**Mechanical power (MP)**	Mechanical power (MP) provided by the ventilator in the pressure-controlled mode was calculated using VT, respiratory rate (RR), and IPAP [3-5]:<br><br>$$MP\ (J/min) = 0.098 * VT * RR * IPAP$$<br>$$MP\ (J/min) = 0.098 * VE * IPAP$$<br><br>With each breath delivered by the ventilator a certain amount of energy (Joule) is transferred to the patients` respiratory system. This energy is mainly used to overcome resistance of the airways and to inflate the lungs and expand the thoracic cage.
**Mechanical power normalized to predicted body weight (PBW-MP)**	MP normalized to predicted body weight (PBW-MP) provided by the ventilator was calculated using MP and predicted body weight (PBW):<br><br>**PBW-MP (J/min/kg) = MP / PBW**<br><br>Calculation of PBW [6]:<br><br>**PBW (males) = 50 + 0.91 * (body height [cm]–152.4)**<br>**PBW (females) = 45.5 + 0.91 * (body height [cm]–152.4)**

**Mechanical power normalized to dynamic lung-thorax compliance (LTC_{dyn}-MP)**	MP normalized to lung-thorax compliance (LTC_{dyn}-MP) was calculated using MP and dynamic lung-thorax compliance (LTC_{dyn}) [7]:<br><br>$\mathbf{LTC_{dyn}\text{-}MP\ (J/min * cmH_2O/mL) = MP / LTC_{dyn}}$<br>$\mathbf{LTC_{dyn}\text{-}MP\ (J/min * cmH_2O/mL) = (0.098 * VT * RR * IPAP) * (DP / VT)}$<br>$\downarrow$<br>$\mathbf{LTC_{dyn}\text{-}MP\ (cmH_2O^2/min) = RR * IPAP * DP}$<br>$\mathbf{LTC_{dyn}\text{-}MP\ (cmH_2O^2/min) = RR * IPAP * (IPAP - PEEP)}$<br><br>This formula takes into account different effects of a change in respiratory rate, inspiratory pressure, and PEEP (and thus changes in DP) on delivered energy. Increasing RR leads to a linear rise in energy transfer, while an increase in pressure (concomitantly increasing tidal volume) results in an exponential increment in power[3].
**Mechanical power normalized to dynamic lung compliance (C_{lung}-MP)**	Introducing esophageal pressure (P_{es}) to the formula for LTC_{dyn}-MP, thereby replacing IPAP by absolute end-inspiratory transpulmonary pressure ($P_{L\_end\text{-}insp}$) and DP by the transpulmonary driving pressure (ΔP_L), leads to an equation that estimates the MP transferred to the lungs in the pressure-controlled ventilation mode (C_{lung}-MP):<br><br>$\mathbf{C_{lung}\text{-}MP\ (cmH_2O^2/min) = RR * P_{L\_end\text{-}insp} * \Delta P_L}$<br>$\mathbf{C_{lung}\text{-}MP\ (cmH_2O^2/min) = RR * (IPAP - P_{es\_end\text{-}insp}) * ([IPAP - P_{es\_end\text{-}insp}] - [PEEP - P_{es\_end\text{-}exp}])}$<br><br>Provided all other variables remain constant, an increase in RR, in absolute $P_{L\_end\text{-}insp}$ (i.e. when a decrease in P_{es} occurs), or in ΔP_L (i.e. by decreasing PEEP) leads to an increment in MP per ventilated unit of lung volume.
**Power index of the respiratory system (PI_{rs})**	LTC_{dyn}-MP normalized to P_aCO_2 was calculated using LTC_{dyn}-MP and P_aCO_2:<br><br>$\mathbf{PI_{rs}^X\ (cmH_2O^2/min) = LTC_{dyn}\text{-}MP * (P_aCO_{2\text{-}actual}/P_aCO_{2\text{-}target})^X}$<br>$\mathbf{PI_{rs}^X\ (cmH_2O^2/min) = RR * IPAP * DP * (P_aCO_{2\text{-}actual}/P_aCO_{2\text{-}target})^X}$<br><br>Since P_aCO_2 is inversely proportional to minute ventilation (neglecting dead space fraction), exponent X approximates values between 1 and 2, depending on whether adjustments of ventilator settings are made for RR and/or IPAP/DP to reach $P_aCO_{2\text{-}target}$, which was set at 45.0 mmHg (6.0 kPa, corresponding to the hypercapnic threshold) for all patients. PI_{rs} equals to the LTC_{dyn}-MP necessary to provide adequate alveolar ventilation (keeping P_aCO_2 below the hypercapnic threshold).

Ventilator weaning

Weaning included protocol-based increasing periods of unassisted breathing through a tracheostomy collar (weaning trials), usually starting with a 30 min SBT, and then increasing by approximately two hours per day. In the intervals between SBT, all patients were ventilated in the pressure-controlled, assisted-controlled (A/C) mode (Vivo 50/55, Breas Medical AB, Moelnlycke, Sweden) to recover from the imposed work of breathing during SBT, but there was no use of high-flow oxygen therapy.

For these weaning trials, patients were placed in the semi-recumbent position, ventilator variables were recorded, and an arterial blood gas analysis (aBGA) was performed. The patient was then disconnected from the ventilator and breathed room air through a T-piece, with oxygen admixture at the same level as on mechanical ventilation. The first SBT was performed under the supervision of a respiratory therapist, and vital signs were continuously monitored to immediately detect respiratory distress. Another aBGA was performed at the end of the SBT and, if possible, also in the event of premature termination of SBT due to respiratory distress. Failure of the SBT was defined as the occurrence of objective clinical signs of respiratory failure (breathing frequency > 35/min, tachycardia > 130 bpm, systolic blood pressure > 160 mmHg, or S_pO_2 < 88% despite increasing oxygen admixture) and/or changes in blood gas values consistent with ventilatory failure (hypercapnia [P_aCO_2 > 45.0 mmHg] with or without respiratory acidosis [pH < 7.35]) [8].

The overall weaning program included nutritional support, proactive physiotherapy, and optimal therapy of comorbidities, with use of sedatives avoided. Immediately upon admission, swallowing was assessed by a speech therapist and, if necessary, speech therapy was initiated, accompanied by increasing periods of daily cuff deflation.

Statistical analysis – Metrics of diagnostic accuracy

ROC curve analysis

To assess the accuracy of the variables analyzed to predict weaning outcome, a receiver operating characteristic (ROC) curve analysis was performed in the entire study population [Figure S1] and diagnostic performance was expressed as the area under the ROC curve (AUROC). Furthermore, we compared ROC curves of different prediction variables and different subgroups of patients.

k-fold cross-validation

To ensure that the proportion of patients with successful and unsuccessful weaning in both groups reflected the proportion in the whole study population (stratification of outcome), patients were randomly assigned to one of two groups in a stratified, 2 times repeated, 2-fold cross-validation [9] (groups A/B or C/D) [Figure S1]. Each of the two groups then acted once as a training set and once as a test set. Threshold values that best predicted failure of prolonged weaning were derived from the training sets using ROC curve analysis by means of the non-parametric method from DeLong [10]. The thresholds that 1) minimized the difference between sensitivity and specificity (assuming equal clinical implications from a false positive and a false negative test), and 2) resulted in the fewest false classifications (the criterion associated with the Youden index [11]) were then used in the test sets to determine the diagnostic performance of each variable. The resulting cross-validated performance of each index, expressed as sensitivity (Sens), specificity (Spec), positive predictive value (PPV), negative predictive value (NPV), accuracy (ACC), positive likelihood ratio (PLR), negative likelihood ratio (NLR), diagnostic odds ratio (DOR) [12], F_1 score, and Matthews correlation coefficient (MCC) [13], equals to the averaged metrics derived from all (four) test sets [Figure S1, Figure S2].

Figure S1: Statistical methods – ROC curve analysis and prospective 2-times repeated, 2-fold cross validation

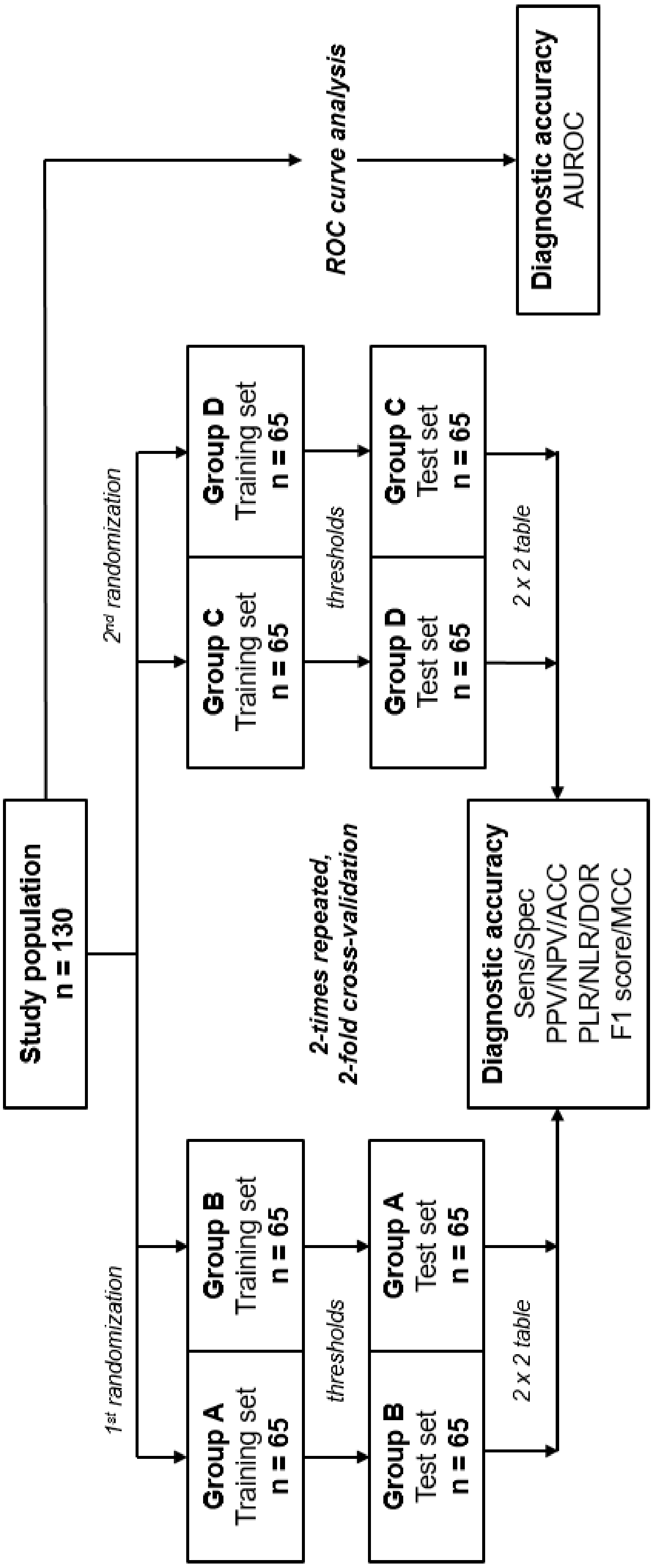

Abbreviations: Sens, sensitivity; Spec, specificity; PPV, positive predictive value; NPV, negative predictive value; ACC, accuracy; PLR, positive likelihood ratio; NLR, negative likelihood ratio; DOR, diagnostic odds ratio; MCC, Matthews correlation coefficient; ROC, receiver operating characteristic (curve); AUROC, area under the ROC curve.

Figure S2: 2 x 2 confusion matrix – Metrics of diagnostic accuracy

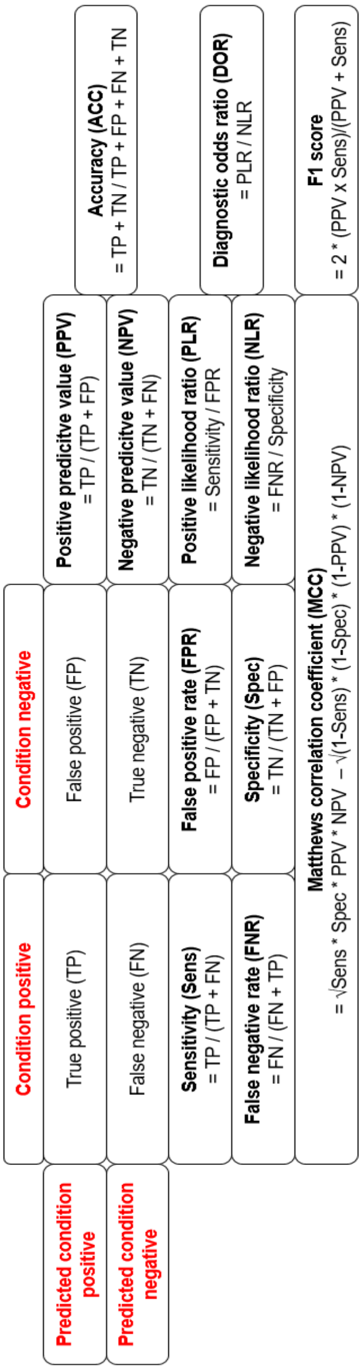

A good performance of a medical diagnostic test usually is defined as [12-13]:

Diagnostic metric	Value	Range
Sensitivity	> 0.80	0.00 – 1.00
Specificity	> 0.80	0.00 – 1.00
Accuracy	> 0.80	0.00 – 1.00
Positive Likelihood Ratio	> 5.0	1.00 – infinity
Negative Likelihood ratio	< 0.20	1.00 – > 0.00
Diagnostic odds ratio	> 10.0	1.00 – infinity
F1 score	> 0.70	0.00 – 1.00
Matthews correlation coefficient	< -0.50 / > 0.50	-1.00 – 1.00
Area under the ROC curve	> 0.80	0.50 – 1.00

Table S1: Clinical characteristics on admission to the weaning center – comparison of groups A/B and C/D

**Clinical characteristics**	**All patients (n = 130)**	**Group A (n = 65)**	**Group B (n = 65)**	**Group C (n = 65)**	**Group D (n = 65)**	***P* value**[a]
Age (years)	69 (60–76)	68 (58–74)	71 (62–76)	67 (59–74)	71 (62–76)	n.s.[b]
Gender (male)	82 (63.1)	43 (66.2)	39 (60.0)	43 (66.2)	39 (60.0)	n.s.[c]
Body mass index (kg/m^2)	27.9 (± 6.9)	27.8 (± 6.6)	27.9 (± 7.3)	29.0 (± 7.5)	26.8 (± 6.1)	n.s.[b]
Obesity (BMI ≥ 30 kg/m^2)	40 (30.8)	18 (27.7)	22 (33.8)	24 (36.9)	16 (24.6)	n.s.[c]
Smoking history	48 (36.9)	23 (35.4)	25 (38.5)	24 (36.9)	24 (36.9)	n.s.[c]
APACHE-II (points)	15.9 (± 5.3)	15.4 (± 5.4)	16.3 (± 5.1)	15.8 (± 5.2)	15.9 (± 5.4)	n.s.[b]
Albumin (g/dL)	2.2 (± 0.5)	2.2 (± 0.6)	2.2 (± 0.4)	2.2 (± 0.5)	2.2 (± 0.5)	n.s.[b]
VD on admission (days)	25 (16–34)	25 (16–34)	25 (17–35)	26 (16–33)	25 (16–35)	n.s.[b]
ETI to tracheostomy (days)	12 (7–18)	12 (8–18)	11 (6–17)	12 (7–18)	11 (7–17)	n.s.[b]
ECLA	14 (10.8)	8 (12.3)	6 (9.2)	9 (13.8)	5 (7.7)	n.s.[c]
**Reason for MV**						
Pneumonia	51 (39.2)	25 (38.5)	26 (40.0)	27 (41.5)	24 (36.9)	n.s.[c]
Surgery	32 (24.6)	20 (30.8)	12 (18.5)	12 (18.5)	20 (30.8)	n.s.[c]
Cardiopulmonary resuscitation	10 (7.7)	5 (7.7)	5 (7.7)	4 (6.2)	6 (9.2)	n.s.[c]
Acute exacerbation of COPD	10 (7.7)	4 (6.2)	6 (9.2)	4 (6.2)	6 (9.2)	n.s.[c]
Sepsis (including septic shock)	7 (5.4)	2 (3.1)	5 (7.7)	5 (7.7)	2 (3.1)	n.s.[d]
Acute heart failure	6 (4.6)	3 (4.6)	3 (4.6)	4 (6.2)	2 (3.1)	n.s.[d]
Other	17 (13.1)	6 (9.2)	11 (16.9)	10 (15.4)	7 (10.8)	n.s.[c]
**Comorbidities**						
Charlson comorb. index (points)	5.5 (± 2.3)	5.4 (± 2.5)	5.6 (± 2.1)	5.3 (± 2.2)	5.6 (± 2.4)	n.s.[b]
Renal insufficiency	46 (35.4)	21 (32.3)	25 (38.5)	25 (38.5)	21 (32.3)	n.s.[c]
Hemodialysis	24 (18.5)	11 (16.9)	13 (20.0)	15 (23.1)	9 (13.8)	n.s.[c]
Diabetes mellitus	35 (26.9)	21 (32.3)	14 (21.5)	20 (30.8)	15 (23.1)	n.s.[c]
Coronary artery disease	33 (25.4)	16 (24.6)	17 (26.2)	12 (18.5)	21 (32.3)	n.s.[c]
COPD	30 (23.1)	12 (18.5)	18 (27.7)	14 (21.5)	16 (24.6)	n.s.[c]
Chronic heart failure	17 (13.1)	5 (7.7)	12 (18.5)	10 (15.4)	7 (10.8)	n.s.[c]
Malignancy	10 (7.7)	***8 (12.3)***	***2 (3.1)***	6 (9.2)	4 (6.2)	***0.049***[c]
Hepatopathy	7 (5.4)	3 (4.6)	4 (6.2)	4 (6.2)	3 (4.6)	n.s.[d]
Interstitial lung disease	8 (6.2)	3 (4.6)	5 (7.7)	4 (6.2)	4 (6.2)	n.s.[d]

Legend

Continuous variables are presented as arithmetic means (± standard deviation) or median (– interquartile range [IQR]); categorical variables are presented as number (%).

a: P value for differences between patients in groups A/B and groups C/D; n.s. [not significant] indicates that there was a significant difference neither between groups A and B, nor between groups C and D
b: Mann-Whitney *U*-test
c: Chi-squared test
d: Fisher`s exact test

Table S2: Results of prolonged weaning – comparison of patients with weaning success and weaning failure

Results of prolonged weaning	All patients (n = 130)	Weaning success (n = 86)	Weaning failure (n = 44)	*P* value[a]
Time from admission to first SBT (days)	1 (0–3)	1 (0–2)	2 (0–4)	0.968[b]
Weaning duration from first SBT (days)	12 (10–17)	12 (10–14)	14 (12–23)	**0.002[b]**
Duration of mechanical ventilation (days)	42 (31–55)	42 (30–55)	42 (34–56)	0.834[b]
Median P_aCO_2 at completion (mmHg)	42.6 (± 7.2)	38.3 (± 4.1)	50.9 (± 3.8)	–
Ventilator-attached on discharge	43 (33.1)	1 (1.2)	42 (95.4)	–
HMV-NIV	20 (15.4)	1 (1.2)	19 (43.1)	–
HMV-IMV	23 (17.7)	0 (0.0)	23 (52.3)	–
SB on discharge (hours per day)*	24 (16–24)	24 (24–24)	14 (4–18)	**< 0.001[b]**
LTOT on discharge§	89 (68.5)	48 (55.8)	41 (93.2)	**< 0.001[c]**
Weaning unit-LOS (days)	29 (22–42)	27 (20–36)	36 (28–49)	**< 0.001[b]**
Hospital-LOS (days)	36 (27–51)	34 (24–49)	43 (29–54)	**0.031[b]**

Legend

Continuous variables are presented as arithmetic means (± standard deviation) or median (– interquartile range [IQR]); categorical variables are presented as number (%).

*: Values for deceased patients after weaning completion were set at 0 hours
§: Excluding two patients with weaning success and three patients with weaning failure, who died after weaning completion

a: P value for differences between patients with weaning success and weaning failure
b: Mann-Whitney *U*-test
c: Chi-squared test

Table S3: Cross-validated performance of variables derived from the mechanical power analyzed to predict weaning outcome – mean values derived from the test sets

**Variables** (threshold)	**Failure of prolonged weaning**									
	Post-SBT									
	**Sens**	**Spec**	**PPV**	**NPV**	**Accuracy**	**PLR**	**NLR**	**DOR**	**F_1**	**MCC**
**MP** (21.2 J/min)	61 (40-79)	67 (51-80)	48 (34-61)	79 (67-86)	65 (52-76)	1.8 (1.1-3.1)	0.6 (1.0-0.3)	4.4	0.53	0.27
**PBW-MP** (0.3609 J/min/kg)	58 (37-77)	81 (66-91)	61 (42-76)	80 (70-86)	73 (61-78)	3.3 (1.6-7.2)	0.5 (0.9-0.3)	8.5	0.58	0.39
**LTC_{dyn}-MP** (7775 cmH_2O^2/min)	66 (45-83)	70 (56-82)	56 (41-69)	82 (69-89)	69 (56-80)	2.7 (1.4-5.4)	0.4 (0.9-0.3)	8.1	0.59	0.37
**$PI_{rs}^{1.0}$** (4740 cmH_2O^2/min)	74 (54-87)	61 (46-74)	51 (39-63)	86 (70-92)	65 (53-77)	2.1 (1.3-3.7)	0.4 (0.9-0.2)	10.4	0.58	0.36
**$PI_{rs}^{2.0}$** (3676 cmH_2O^2/min)	78 (57-92)	65 (50-77)	57 (44-67)	86 (70-93)	69 (57-80)	3.0 (1.6-5.6)	0.3 (0.8-0.1)	10.5	0.64	0.43
**Variables** (threshold)	*Pre-weaning completion*									
	**Sens**	**Spec**	**PPV**	**NPV**	**Accuracy**	**PLR**	**NLR**	**DOR**	**F_1**	**MCC**
**MP** (19.3 J/min)	86 (65-97)	56 (40-71)	50 (41-60)	89 (73-96)	66 (53-77)	2.0 (1.4-3.0)	0.2 (0.7-0.1)	9.0	0.64	0.41
**PBW-MP** (0.3148 J/min/kg)	67 (45-84)	63 (47-77)	48 (36-61)	80 (67-88)	64 (51-76)	1.8 (1.1-3.1)	0.5 (1.0-0.3)	4.0	0.55	0.29
**LTC_{dyn}-MP** (6047 cmH_2O^2/min)	79 (59-92)	74 (58-86)	60 (47-73)	88 (76-94)	76 (64-85)	3.2 (1.8-5.6)	0.3 (0.6-0.1)	21.2	0.68	0.51
**$PI_{rs}^{1.0}$** (4778 cmH_2O^2/min)	82 (62-93)	80 (65-90)	68 (53-80)	90 (78-95)	81 (69-89)	4.3 (2.2-8.4)	0.2 (0.5-0.1)	42.6	0.74	0.60
**$PI_{rs}^{2.0}$** (3896 cmH_2O^2/min)	81 (59-94)	87 (72-95)	76 (59-88)	90 (79-95)	85 (74-93)	6.7 (2.9-16)	0.2 (0.5-0.1)	42.5	0.78	0.67

Legend

Assessment of mean sensitivity and specificity, positive and negative predictive value, positive and negative likelihood ratio, diagnostic odds ratio, F_1 score, and Matthews correlation coefficient (with 95% confidence intervals) based on threshold values associated with the *Youden index*.

Table S4: Ventilator variables and mechanical power indices predicting the outcome of prolonged weaning – comparison of patients with weaning success and weaning failure

**Variables** *(48 h post-SBT)*	**All patients (n = 130)**	**Weaning success (n = 86)**	**Weaning failure (n = 44)**	***P* value**[a]
**No. of observations (per patient)**	5.4	–	–	–
**RR (1/min)**	17.4 (± 2.3)	17.2 (± 2.4)	17.8 (± 2.1)	0.614[c]
**VTi (mL)**	545 (± 78)	546 (± 76)	543 (± 84)	0.887[c]
**VE (L/min)**	9.5 (± 1.7)	9.4 (± 1.7)	9.6 (± 1.8)	0.517[c]
**P_aCO_2 on MV (mmHg)**	35.0 (± 5.4)	33.0 (± 3.9)	38.9 (± 5.9)	**< 0.001**[c]
**VR**	1.26 (± 0.34)	1.14 (± 0.23)	1.49 (± 0.40)	**< 0.001**[c]
**IPAP (cmH_2O)**	22.5 (± 4.1)	21.0 (± 3.3)	25.4 (± 4.0)	**< 0.001**[c]
**DP (cmH_2O)**	16.5 (± 3.9)	15.1 (± 3.1)	19.3 (± 3.9)	**< 0.001**[b]
**LTC_{dyn} (mL/cmH_2O)**	35.0 (± 10.7)	37.8 (± 10.6)	29.5 (± 8.1)	**< 0.001**[c]
**MP (J/min)**	21.1 (± 5.5)	19.6 (± 4.6)	24.1 (± 5.8)	**< 0.001**[b]
**PBW-MP (J/min/kg)**	0.3311 (± 0.1053)	0.2958 (± 0.0750)	0.4002 (± 0.1213)	**< 0.001**[c]
**LTC_{dyn}-MP (cmH_2O^2/min)**	6847 (± 3162)	5738 (± 2386)	9013 (± 3388)	**< 0.001**[c]
**$PI_{rs}^{1.0}$ (cmH_2O^2/min)**	5478 (± 3258)	4245 (± 2062)	7890 (± 3801)	**< 0.001**[c]
**$PI_{rs}^{2.0}$ (cmH_2O^2/min)**	4561 (± 3656)	3187 (± 1853)	7247 (± 4707)	**< 0.001**[c]
**Variables** *(48 h pre-weaning completion)*	**All patients (n = 130)**	**Weaning success (n = 86)**	**Weaning failure (n = 44)**	***P* value**[a]
**No. of observations (per patients)**	2.8	–	–	–
**RR (1/min)**	17.4 (± 2.7)	17.3 (± 2.9)	17.6 (± 2.2)	0.768[c]
**VTi (mL)**	557 (± 95)	565 (± 98)	540 (± 87)	0.503[c]
**VE (L/min)**	9.8 (± 2.3)	9.9 (± 2.5)	9.5 (± 1.9)	0.527[c]
**P_aCO_2 on MV (mmHg)**	35.0 (± 5.3)	32.8 (3.4)	39.4 (5.6)	**< 0.001**[b]
**VR**	1.29 (± 0.35)	1.18 (± 0.28)	1.49 (± 0.40)	**< 0.001**[c]
**IPAP (cmH_2O)**	21.8 (± 4.0)	20.1 (± 3.1)	25.1 (± 3.6)	**< 0.001**[b]
**DP (cmH_2O)**	16.1 (± 3.8)	14.5 (± 3.0)	19.2 (± 3.4)	**< 0.001**[b]
**LTC_{dyn} (mL/cmH_2O)**	37.2 (± 14.2)	41.2 (± 15.2)	29.3 (± 6.9)	**< 0.001**[c]
**MP (J/min)**	20.9 (± 6.2)	19.6 (± 5.9)	23.5 (± 6.0)	**< 0.001**[c]
**PBW-MP (J/min/kg)**	0.3271 (± 0.1083)	0.2955 (± 0.0879)	0.3888 (± 0.1184)	**< 0.001**[c]
**LTC_{dyn}-MP (cmH_2O^2/min)**	6489 (± 3098)	5325 (± 2391)	8762 (± 3083)	**< 0.001**[c]
**$PI_{rs}^{1.0}$ (cmH_2O^2/min)**	5179 (± 3095)	3873 (± 1849)	7734 (± 3447)	**< 0.001**[c]
**$PI_{rs}^{2.0}$ (cmH_2O^2/min)**	4219 (± 3251)	2841 (± 1508)	6912 (± 4000)	**< 0.001**[c]

Legend

Continuous variables are presented as arithmetic means values (± standard deviation).

a: P value for differences between patients with success and failure of the SBT or prolonged weaning
b: Student`s *t*-test
c: Mann-Whitney *U*-test

Figure S3: ROC curves for selected variables and mechanical power indices derived from the entire study population (n = 130) 48 hours before weaning completion

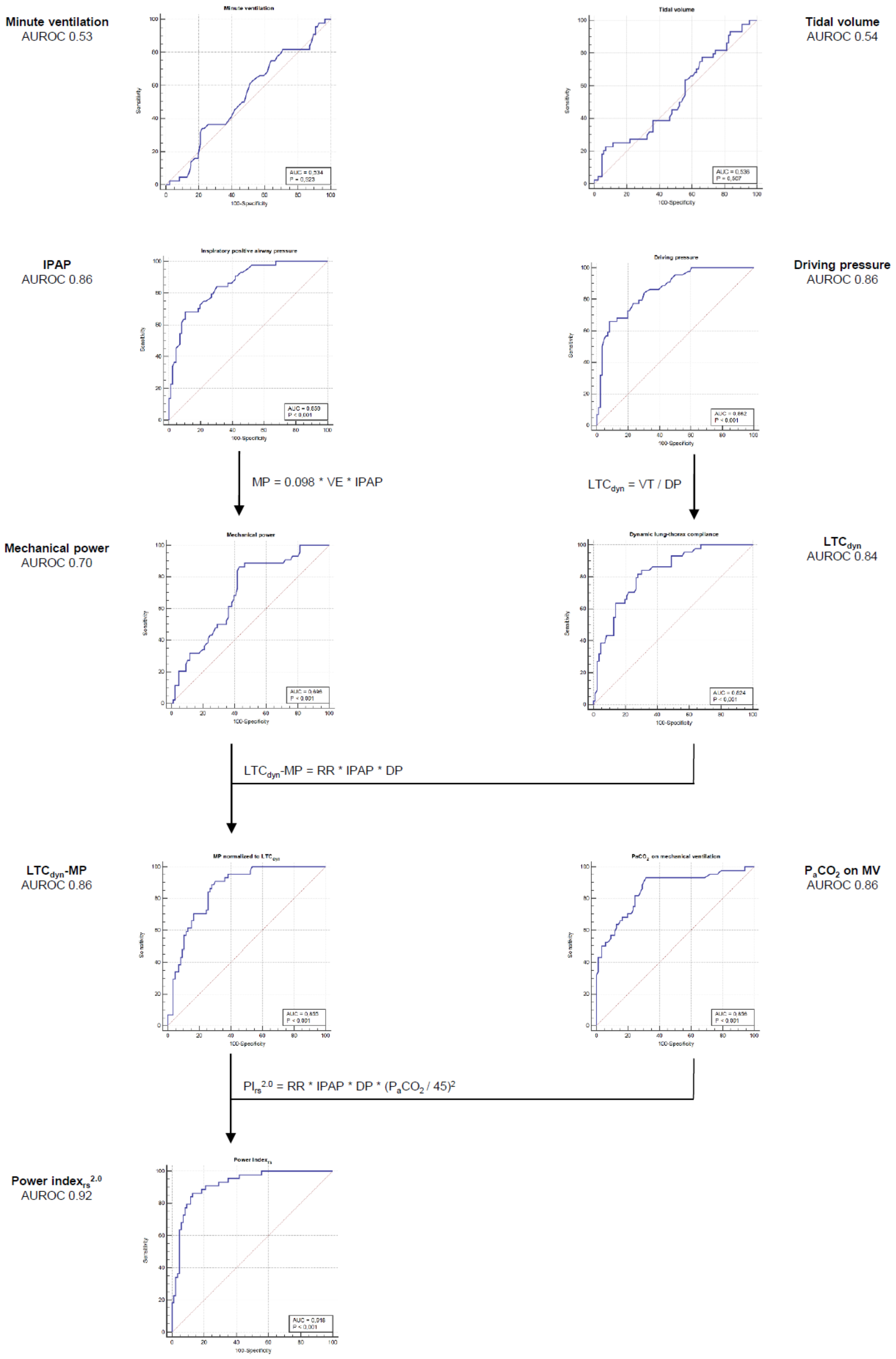

Abbreviation list

- aBGA: Arterial blood gas analysis
- ACC: Accuracy
- APACHE-II: Acute Physiology and Chronic Health Evaluation II score
- AUROC: Area under the receiver operating characteristic curve
- BMI: Body mass index
- COPD: Chronic obstructive pulmonary disease
- DOR: Diagnostic odds ratio
- DP: Driving pressure
- ECLA: Extracorporeal lung assistance
- ETI: Endotracheal intubation
- F_1: F_1 score
- F_iO_2: Fraction of inspired oxygen
- HMV-IMV: Home mechanical ventilation–invasive mechanical ventilation
- HMV-NIV: Home mechanical ventilation–non-invasive mechanical ventilation
- IMV: Invasive mechanical ventilation
- IPAP: Inspiratory positive airway pressure
- LOS: Length of stay
- LTC_{dyn}: Dynamic lung-thorax compliance
- LTC_{dyn}-MP: MP normalized to dynamic lung-thorax compliance
- LTOT: Long-term oxygen therapy
- MCC: Matthews correlation coefficient
- MP: Mechanical power
- MV: Mechanical ventilation
- NLR: Negative likelihood ratio

- No.: Number
- NPV: Negative predictive value
- PBW-MP: MP normalized to predicted body weight
- PI_{rs}: Power index of the respiratory system
- PLR: Positive likelihood ratio
- PPV: Positive predictive value
- ROC: Receiver operating characteristic (curve)
- RR: Respiratory rate
- SB: Spontaneous breathing
- SBT: Spontaneous breathing trial
- Sens: Sensitivity
- Spec: Specificity
- VE: Minute ventilation
- VR: Ventilatory ratio
- VTi: Inspiratory tidal volume

References

1. Sinha P, Fauvel NJ, Singh S, et al: Ventilatory ratio: a simple bedside measure of ventilation. *Br J Anaesth* 2009;102:692–97
2. Sinha P, Fauvel NJ, Singh P, et al: Analysis of ventilatory ratio as a novel method to monitor ventilatory adequacy at the bedside. *Crit Care* 2013;17:R34
3. Gattinoni L, Tonetti T, Cressoni M, et al: Ventilator-related causes of lung injury: the mechanical power. *Intensive Care Med* 2016;42:1567–1575
4. Becher T, van der Staay M, Schädler D, et al: Calculation of mechanical power for pressure-controlled ventilation. *Intensive Care Med* 2019;45:1321–23
5. Chiumello D, Gotti M, Guanziroli M, et al: Bedside calculation of mechanical power during volume- and pressure controlled mechanical ventilation. *Crit Care* 2020;24:417
6. Brower RG, Matthay MA, Morris A, et al: Ventilation with lower tidal volumes as compared with traditional tidal volumes for acute lung injury and the acute respiratory distress syndrome. *N Engl J Med* 2000;342: 1301–1308
7. Ghiani A, Paderewska J, Sainis A, et al: Variables predicting weaning outcome in prolonged mechanically ventilated tracheotomized patients: a retrospective study. *J Intensive Care* 2020;8:19
8. Boles JM, Bion J, Connors A, et al. Weaning from mechanical ventilation. *Eur Respir J* 2007;29(5):1033–1056
9. Arlot S, Celisse A: A survey of cross-validation procedures for model selection. *Stat Surv* 2010;4:40–79
10. DeLong ER, DeLong DM, Clarke-Pearson DL: Comparing the areas under two or more correlated receiver operating characteristic curves: a nonparametric approach. *Biometrics* 1988;44:837–845
11. Youden WJ: Index for rating diagnostic tests. *Cancer* 1950;3:32–35

12. Fischer JE, Bachmann LM, Jaeschke R. A readers` guideline to the interpretation of diagnostic test performance: clinical example of sepsis. *Intensive Care Med.* 2003;29:1043–1051
13. Chicco D, Jurman G: The advantages of the Matthews correlation coefficient (MCC) over F1 score and accuracy in binary classification evaluation. *BMC Genomics.* 2020;21:6
